# Trust in Science: CRISPR–Cas9 and the Ban on Human Germline Editing

**DOI:** 10.1007/s11948-017-9931-1

**Published:** 2017-06-26

**Authors:** Stephan Guttinger

**Affiliations:** Egenis, Centre for the Study of Life Sciences, University of Exeter, Byrne House, St German’s Road, Exeter, EX4 4PJ UK

**Keywords:** CRISPR–Cas9, Genome editing, Human embryo, Moratorium, Asilomar conference, Recombinant DNA technology

## Abstract

In 2015 scientists called for a partial ban on genome editing in human germline cells. This call was a response to the rapid development of the CRISPR–Cas9 system, a molecular tool that allows researchers to modify genomic DNA in living organisms with high precision and ease of use. Importantly, the ban was meant to be a trust-building exercise that promises a ‘prudent’ way forward. The goal of this paper is to analyse whether the ban can deliver on this promise. To do so the focus will be put on the precedent on which the current ban is modelled, namely the Asilomar ban on recombinant DNA technology. The analysis of this case will show (a) that the Asilomar ban was successful because of a specific two-step containment strategy it employed and (b) that this two-step approach is also key to making the current ban work. It will be argued, however, that the Asilomar strategy cannot be transferred to human genome editing and that the current ban therefore fails to deliver on its promise. The paper will close with a reflection on the reasons for this failure and on what can be learned from it about the regulation of novel molecular tools.

## Introduction

The molecular life sciences are an area of research that constantly creates new opportunities for human intervention in biological systems. A prominent example of such an intervention is the manipulation of genomic DNA, a topic that has received increased attention in recent years. Responsible for this increase in attention is a new technology that allows to modify genomic DNA in almost any organism with high efficiency, precision, and ease of use, the so-called CRISPR–Cas9 system (Jinek et al. 2012; Ledford 2015).

This new molecular tool is quickly taking over and transforming the field of genome editing (Pennisi 2013). Importantly, with the advent of this tool the editing of living human embryos is rapidly becoming a reality: in February 2016 the first research project in the UK to modify the genomes of human embryos using CRISPR–Cas9 has been approved by the Human Fertilisation and Embryology Authority (HFEA) (Callaway 2016). Before that, researchers in China already applied the method to (non-viable) human embryos (Liang et al. 2015).^1^

The method, however, is not perfect and there is still considerable uncertainty surrounding its application, especially when it comes to (a) the precision and (b) the effects of the DNA modifications it allows researchers to make. This uncertainty—even though significantly smaller than with older methods—is problematic because the manipulation of genomic DNA is a potentially powerful intervention; changing the DNA of an organism can not only have beneficial but also serious negative effects on the development and/or health of the modified individual (or the environment more generally).

Scientists are aware of the system’s problems and have therefore called for a self-imposed ban on certain uses of CRISPR–Cas9 until the risks involved have been further analysed (Baltimore et al. 2015; Lanphier et al. 2015). The ban, which will be discussed in more detail in “CRISPR–Cas9, the Germline, and Uncertainty” section, in particular calls for a temporary suspension of germline genome editing in humans (for clinical use) and for further evaluation of the efficacy and specificity of the CRISPR–Cas9 system (see Baltimore et al. 2015).

Importantly, this ban is more than just a precautionary measure. As Jasanoff et al. (2015) and Sarewitz (2015) point out, it is also an exercise in trust-building: part of the idea behind the self-imposed restrictions is to demonstrate to the larger public (including policy-makers) that scientists are behaving in an ethically responsible manner, putting breaks on their own work when necessary and thereby making additional outside regulation superfluous. The promise here is that scientists are moving forward in a “prudent” way (Baltimore et al. 2015), following a path that is both powerful *and* safe.

There are, however, several issues such a prudent way forward faces. For one there is the question of how risk is defined in the first place. Who gets to decide what the relevant risks are and how they have to be tackled? As several authors have pointed out, the definition of the risks of scientific research (and its products) is often done by scientists themselves without the inclusion of a broader group of stakeholders. This can lead to a rather narrow definition of the risks involved, a problem that could be overcome by using a more inclusive and democratic approach to defining and dealing with risk (Jasanoff et al. 2015; Sarewitz 2015).

But there is also a second question that comes up here, namely whether the measures that *were* proposed (irrespective of who has proposed them) can actually achieve what they promise to do. Answering this question is important as the trustworthiness of the way forward depends on these measures and their success. Even though most people will accept that no process is 100% safe, if a strategy fails to deliver then the prudence and trustworthiness of the path forward will be questioned. Importantly, this can also feed into and reinforce a more general mistrust of science, an issue that has become more urgent again in recent years.

The goal of this paper is to address this second question, i.e. to analyse whether the proposed ban on genome editing is an effective measure that can ultimately deliver on its promises. The aim is not only to understand this particular and important instance of a trust-building measure but to also understand more generally how appropriate measures to build (or maintain) public trust in science can and should be developed.

In “CRISPR–Cas9 and the Genetic Modification of Human Embryos” section, the CRISPR–Cas9 system will be introduced in more detail. This will be followed by a discussion of the proposed ban and the reasons behind it (“CRISPR–Cas9, the Germline, and Uncertainty” section). A particular focus will be put on the uncertainties that surround the use of the CRISPR–Cas9 system, as these are key drivers behind the ban. However, it will be argued that in order to fully understand the nature and the structure of the ban it will also be important to look at the precedent on which it is modelled, namely the Asilomar ban on recombinant DNA technology. The analysis of the Asilomar case (“The Asilomar Case” section) will uncover a specific two-step approach that was employed to allow researchers to eventually revise or even lift the ban in a safe manner. “CRISPR–Cas9, Genome Editing and the Two-Step Asilomar Approach” section will illustrate how this two-step approach is also applied to the CRISPR–Cas9 case and how it is crucial for making the current ban work. It will be argued, however, that this transfer ultimately fails as the key conditions that make it work in the Asilomar case are not fulfilled in the CRISPR–Cas9 case. The current ban therefore fails to deliver on its promises. Importantly, this failure only comes to the fore once we take the processual nature of organisms into account. “Conclusion” section will reflect on what this failure means for how the regulation of new molecular tools used in the life sciences and in biomedicine can or should be approached.

## CRISPR–Cas9 and the Genetic Modification of Human Embryos

Ever since the manipulation of DNA in biological systems became a possibility in the 1970s the technology has been in the spotlight of both academic and public discussions. There are spikes in the amount of attention the field gets, which usually correlate with the development/announcement of new methods and projects. One such spike could be observed in the early 1990s when the first gene therapy trials were conducted.^2^ In recent years a new spike in attention could be observed, this time triggered by the fast emergence of the CRISPR–Cas9 system and the possibilities it offers for editing genomic DNA in living cells.

### The CRISPR–Cas9 System

The CRISPR–Cas9 system is a molecular tool that allows researchers to edit DNA in living cells with high precision (Jinek et al. 2012; Cong et al. 2013; Jinek et al. 2013; Mali et al. 2013). The two components of the system—CRISPR and Cas9—are part of a naturally occurring adaptive immune system in bacteria and archaea (Rath et al. 2015). The term ‘CRISPR’ stands for ‘clustered regularly interspaced short palindromic repeats’ and refers to particular sequence motives in bacterial DNA that were first discovered in 1987 (Ishino et al. 1987).^3^ The term ‘Cas’ stands for ‘CRISPR-associated’ and refers to a class of bacterial proteins that include nucleases, i.e. proteins that can mediate the cutting of the DNA double helix.^4^

The function of CRISPRs in bacterial DNA has been elusive for a long time and only about 20 years after their discovery researchers had collected enough data to suggest that they might form part of an adaptive immune system in bacteria and archaea (a hypothesis confirmed by Makarova et al. 2006). A key finding in this context was that CRISPRs contain short sequences that correspond to DNA found in bacteriophages, the viruses that can infect bacterial cells (Mojica et al. 2005; Pourcel et al. 2005; Bolotin et al. 2005). These CRISPR sites are transcribed into RNA molecules that then form an intracellular complex with different Cas proteins (Lillestøl et al. 2006; Brouns et al. 2008). The role of the RNA is to guide the complex to the bacteriophage DNA, which the Cas nuclease in the complex then cuts at the site specified by the CRISPR-derived RNA. This cleavage inhibits the generation of further bacteriophage particles, a process that depends on intact bacteriophage DNA.^5^

What is probably most astonishing about the CRISPR-Cas system is how easily it can be adapted as a molecular tool in the laboratory. Especially when using the Cas9 protein as the nuclease of choice, the CRISPR system provides researchers with a simple method for creating organisms with an altered genome. One way of putting this system to use is by injecting the mRNA coding for Cas9 and a specially designed guide RNA into the fertilized egg (zygote) of the target organism (or any other cell the researcher wishes to modify).^6^ The guide RNA is designed to mimic natural CRISPR-derived RNAs and contains (a) key structural features that allow the RNA to form a complex with the Cas9 nuclease and (b) parts that are complementary to the DNA sequence of choice. The injected cell will use the mRNA to produce the Cas9 protein, which then forms a complex with the injected guide RNA. This complex will then be targeted to the DNA sequence of interest. After the complex binds to the target site the nuclease cuts the genomic DNA and thereby triggers cellular repair processes that can be exploited by the researcher to modify the genomic sequence (for a review of these editing mechanisms see Sander and Joung 2014).^7^

### What is New About CRISPR–Cas9?

Even though the modification of human genomes has been possible since the development of gene therapy in the 1980s (see footnote 2), there are some key differences between traditional gene therapy and the genome editing that CRISPR–Cas9 allows for. First, traditional gene therapy techniques (mostly using retroviral systems for the delivery of DNA to target cells) do not offer any control over where in the genome the DNA modification happens. The viral vector that is used will insert its DNA construct at more or less random sites. As the random insertion of additional DNA into a genome can lead to unintended consequences (Biasco et al. 2012), gene therapy carries a risk element that the CRISPR–Cas9 system does not suffer from (at least not to the same degree, see “CRISPR–Cas9 and Uncertainty” section). Second, standard gene therapy only allows researchers to *add* DNA segments to a genome, i.e. to insert, for instance, an extra copy of a gene. The CRISPR–Cas9 system, in contrast, allows researchers to also delete and/or replace specific sites with other sequences and therefore greatly expands the range of interventions that are possible.^8^

It has to be mentioned here that the targeted editing of genomes has been possible for a number of years now through the use of zinc-finger nucleases (ZFNs) and ‘transcription activator-like effector nucleases’ (TALENs) (Gaj et al. 2013). What these earlier methods lack, however, is the ease and efficiency the CRISPR–Cas9 system offers, as in contrast to CRISPR–Cas9 the ZFN and TALEN systems use protein modules to target a nuclease to the genomic DNA. This means that the researchers have to develop specific sequence-targeting proteins for each new application of the system, a step that is much more labour-intensive and error-prone than the RNA-based approach that the CRISPR–Cas9 system offers. The revolution that the CRISPR–Cas9 systems brings about is therefore mainly one of reduced effort and cost. This makes it possible for researchers to do experiments that before were extremely difficult to do and also explains, in part at least, the rapid and broad uptake the technology has found in the research community (Pennisi 2013; Baltimore et al. 2015).

## CRISPR–Cas9, the Germline, and Uncertainty

Even though the CRISPR–Cas9 system allows researchers to modify almost any cell type from any organism, the central issue that stands out in the current discussions about the system is the editing of genomic DNA in human embryos. A key reason the editing of embryo genomes is so controversial is that it can lead to the editing of germline cells, which entails the possibility of passing on (potentially harmful) genetic modifications to future generations (something that does not happen if somatic (i.e. non-reproductive) cells are targeted). As the CRISPR–Cas9 system is the one tool that now makes such powerful interventions an accessible and affordable reality, the discussion about germline editing, embryos and CRISPR–Cas9 have become intimately intertwined.

Clearly there are many pressing issues that need to be addressed before germline genome editing and a tool like the CRISPR–Cas9 system can be rolled out on a broader basis [not only the above-mentioned discussions about the definition of the risks involved but also, for instance, questions of consent (Smolenski 2015; Sugarman 2015)]. However, the one thing that is currently dominating the discussions about the new technology and its uses is the question of its safety, an issue that seems to put discussions about broader ethical and social concerns on hold, at least within the scientific community. What is driving this emphasis on safety are the significant uncertainties that still surround the application of the CRISPR–Cas9 system.

### CRISPR–Cas9 and Uncertainty

There are (at least) two aspects of the CRISPR–Cas9 system that are loaded with uncertainty: first, there is uncertainty about whether scientists can actually achieve the DNA manipulations they want to make with enough precision.^9^ The problem here is that, in theory, only the site specified by the guide RNA should be modified when using CRISPR–Cas9. However, even though the system represents a great improvement over earlier methods (that either had no guidance (traditional virus-based gene therapy) or that used proteins as guides (ZNFs or TALENs)) the system is not perfect. A key issue is that the sequence-specific binding of RNA to DNA does not require a perfect match between the two sequences, meaning that a stable (or stable enough) RNA:DNA complex can also be formed with DNA sequences that are similar but not identical to the sequence specified in the guide RNA.^10^ And since the formation of a stable complex seems to be sufficient for a modification to take place the precision of the RNA-based targeting mechanism is a key topic that researchers are now addressing.^11^

The second uncertainty that still surrounds the system has to do with the question of whether a particular manipulation (even if it happens with 100% precision) has the effect on the target organism that it is supposed to have [researchers speak of “on-target events that have unintended consequences” (Baltimore et al. 2015, p. 37)]. This uncertainty has less to do with the CRISPR–Cas9 system itself and more with the consequences of the actual act of modifying genomic DNA in living cells.

An example of such unintended consequences of on-target interventions would be a change in the expression of gene B when the goal was to change the expression of gene A. This could happen, for instance, if researchers unknowingly target a DNA segment that plays several functional roles at once (in this case a DNA sequence that is not only involved in the regulation of gene A but also affects gene B). To reduce this type of uncertainty about the effects of genome modifications the researchers therefore need to (1) know more about the role(s) existing DNA elements play within the cell/organism and (2) be able to predict the behaviour of newly introduced DNA sequences in concrete cellular contexts, two tasks that are still exceedingly difficult to achieve (see “CRISPR–Cas9, Genome Editing and the Two-Step Asilomar Approach” section).

### A Temporary and Partial Ban on Genome Editing in Humans

In January 2015 a small one-day meeting took place in Napa where 18 people—mainly biologists and a couple of other stakeholders—discussed the challenges that the CRISPR–Cas9 system and its uncertainties bring with it (Doudna 2015). In March and April 2015 two papers were published in *Nature* and *Science* that called for a voluntary ban on the use of CRISPR–Cas9 for modifications of the genome of human germline cells in clinical applications (Baltimore et al. 2015; Lanphier et al. 2015). These initial discussions and calls for action (or inaction) were then followed by a three-day conference in Washington D.C. in December 2015, the “International Summit on Human Gene Editing”, where almost 500 researchers and other stakeholders further discussed the issues surrounding the use of CRISPR–Cas9 (and genome editing more generally) (Reardon 2015).^12^

The basic idea of a ban on certain uses of the technology was articulated by Baltimore and colleagues, who stated that steps should be taken to “strongly discourage […] any attempts at germline genome modification for clinical application in humans” and to “[e]ncourage and support transparent research to evaluate the efficacy and specificity of CRISPR–Cas9 genome engineering technology in human and nonhuman model systems relevant to its potential applications for germline gene therapy” (Baltimore et al. 2015, p. 37).^13^ The authors further recommended that forums should be created for (a) the education of stakeholders and the wider public about the technology and (b) the discussion of the ethical, legal and social issues it raises.

That the above-discussed uncertainties surrounding the CRISPR–Cas9 system were a key driver behind the call for a ban becomes clear when the authors state that “[at] present, the potential safety and efficacy issues arising from the use of this technology [i.e. CRISPR–Cas9] must be thoroughly investigated and understood before any attempts at human engineering are sanctioned, if ever, for clinical testing” (ibid., p. 37).^14^

It is important to point out here that the proposed ban is both partial and temporary. The ban is partial because it still allows researchers to edit the genomes of humans (if somatic cells are targeted) and because it still allows for germline editing in non-human organisms. The ban is also temporary as the researchers suggest that it could be revised at some point, depending on the results of further research into the safety and efficacy issues that surround the technology. This (potentially) temporary nature of the ban is crucial as it ensures that the further development/use of the technology is still an option, meaning the ban can still be part of a ‘way forward’.

Importantly, the ban is a precautionary measure, meaning it can be interpreted as an application of the precautionary principle (PP) (Peters 2015). The PP is a principle that is difficult to define (Freestone and Hey 1996) but the general idea behind it is that restrictive measures should be taken regarding activities or entities that could cause harm to humans or the environment, even if there is no proven (usually interpreted as: scientific) link between the activity/entity and its alleged potential for causing harm.^15^ The PP is usually invoked when scientific uncertainty and the possibility of creating irreversible and/or severe damage come together (Myhr and Traavik 2002).

What is important about the PP are two things: (1) the PP shifts the burden of proof to those who want to perform an activity or use an entity without the precautionary restrictions, meaning that they have to find ways of understanding what the actual risk is that the activity/entity poses. Only if such evidence can be provided could the restrictions be revised or even lifted. This also means (2) that the measures are, in principle at least, temporary: if scientists can determine the actual risk potential of the entity or activity of interest, the restrictions could be revised accordingly.

### Dealing with Precautionary Measures

To be able to do research that could be used to revise the precautionary measures it is instrumental that there is a clear separation between the research context and the actual use of the entity or process in question. It is this separation that allows researchers to study the entity or process of interest without risking creating harm (i.e. violating the PP).

An example of such a situation is the precautionary ban on the use of personal electronic devices (PEDs) on airplanes during landing and take-off. The reason for this ban was the fear that signals from these devices could interfere with the electronic systems of an airplane and potentially lead to a fatal crash. Clearly there is potential for great harm here; there was, however, little or no evidence that there is a link between the use of PEDs and the malfunctioning of an airplane’s avionics. The ban has now been lifted^16^ and part of the reason for this is the extensive research that, for instance, airplane manufacturers have conducted since the ban was put in place.^17^ This research included the in-depth analysis of anecdotal reports from airlines about alleged interference events and also ground tests in which airplanes without passengers on board were put through the procedures that are usually at work during take-off and landing to test whether there is indeed any interference from PEDs.

What is crucial here is that it was possible to separate the use and the investigation of the entities and processes of interest. This opened up a safe space within which researchers could investigate potential causal links between the use of PEDs and the interference with the airplane’s systems without having to put people at risk. If no such separation were possible then doing research would mean putting people or the environment at risk, meaning that it would not be possible to further investigate the risks without violating the PP at the same time.

What can be learned from this example for the CRISPR–Cas9 case is that such a separation is also key to realising the promises of the self-imposed ban on genome editing, i.e. the promises that scientists pursue a “prudent way forward” (Baltimore et al. 2015) by investigating and developing the technology in a safe manner; it is the promise of *safe* research that is supposed to make the larger public trust the sciences and its ability to self-regulate. The question is of course if and how researchers can create the safe space for experimentation so they can deliver on their promises. To answer this question the next Section will look at the precedent on which this ban is modelled, namely the Asilomar ban on recombinant DNA technology.

### The Precedent

As mentioned in the introduction, calls for a voluntary ban on emerging technologies have been made before by the scientific community. One example is the call for a temporary ban on the use of recombinant DNA technology in the mid-1970s (Berg et al. 1974) that culminated in the famous Asilomar conference in 1975 (Berg et al. 1975; Fredrickson 1991; Capron and Schapiro 2001).

The Asilomar case is particularly interesting as some of the same scientists who were leading the call back in the 1970s are also involved in the current discussions about CRISPR–Cas9. Importantly, these authors refer to the Asilomar conference as the “original discussions” about the issues involved and thereby put the CRISPR case in a direct line with Asilomar 40 years earlier (Baltimore et al. 2015, 37). This is not surprising as (a) the Asilomar case shows clear analogies to the CRISPR–Cas9 case and (b) it is seen by many as a success story, making it, in principle at least, a powerful precedent (Jasanoff et al. 2015). Importantly, the Asilomar ban was also a case of trust-building as researchers took measures because they “feared that a public debate would place crippling restrictions on molecular biology” (Berg 2008, 291).

The question, however, is what exactly is taken over from the Asilomar case and whether these elements have the same power as they (allegedly) had back then. The next Section will therefore analyze the particular strategy that was proposed at the original Asilomar conference and which was meant to allow researchers to move forward in a safe and responsible manner. “CRISPR–Cas9, Genome Editing and the Two-Step Asilomar Approach” section will then analyze how this strategy is also implemented in the CRISPR–Cas9 case and discuss whether such an implementation can be successful.

## The Asilomar Case

The topic of the Asilomar conference and the discussions that surrounded it was the then-new recombinant DNA technology that allowed researchers to create DNA molecules that contained sequences derived from different sources. The great potential of this technology for both research and industrial applications was not lost on scientists. But it was also clear that the technology had the potential to create great harm, both to humans and the environment more generally. What was at the centre of the discussions at the time was the uncertainty about the behaviour of the recombinant DNA elements and the organisms that carry them.

### Recombinant DNA and Uncertainty

The problem researchers were faced with when dealing with recombinant DNA was that they did not know what would happen if new combinations of DNA sequences (for instance a mix of viral and bacterial DNA) were put together. The fear was that such manipulations could “result in the creation of novel types of infectious DNA elements whose biological properties cannot be completely predicted in advance” (Berg et al. 1974, p. 303). Of particular worry were experiments that would create recombinant DNA containing antibiotic resistance genes, bacterial toxins or sequences from viruses that were known to be able to induce cancer in humans. Importantly, researchers were not only concerned about how the new DNA elements themselves would behave once inserted into organisms (for instance spread uncontrollably to other organisms) but also how the organisms carrying the modified and foreign DNA (i.e. ‘genetically-modified organisms’ or ‘GMOs’) might behave and affect other organisms (for instance modified bacteria that could suddenly become highly pathogenic).

Because of this barely understood potential for harm scientists called for a precautionary ban on experiments with recombinant DNA (Berg et al. 1974), meaning that the Asilomar case can also be interpreted as an application of the PP (Hansson 2016). Importantly, as in the case of CRISPR–Cas9, broader social and ethical issues were put aside in the Asilomar case as safety concerns took centre stage (Capron and Schapiro 2001).^18^

### The Ban

The way in which the discussions about recombinant DNA unfolded in the 1970s is similar to the way the discussions about CRISPR–Cas9 developed (or the other way around): initial informal discussions among peers resulted in the publication of a paper/letter in an academic journal that called for a ban on certain uses of the technology. This initial call was then followed by a larger conference at which concrete ways of dealing with the technology were discussed.

In the Asilomar case a group of scientists—after earlier discussions and smaller gatherings between peers (Fredrickson 1991)—published a letter in 1974 in which they called for a temporary ban on two types of experiments, (1) those that lead to the creation of autonomously replicating DNA plasmids that contain antibiotic resistance genes or bacterial toxins and (2) those that combine DNA from oncogenic and other animal viruses with autonomously replicating DNA elements (of bacterial or viral origin) (Berg et al. 1974). This initial call for a blanket ban on any experiments with such DNA was then refined (and partially lifted) at the Asilomar conference.

### The Two-Step Asilomar Approach and the Idea of Containment

As in the case of PEDs, the problem for those who wanted to use recombinant DNA technology was to figure out how one could perform research on the potential dangers of the technology without having to violate the PP they invoked when calling for a ban. As Philippe Kourilsky, a participant of the Asilomar conference, put it: “On the frontiers of the unknown the analysis of benefits and hazards were locked up in concentric circles of ignorance…how could one determine the reality…without experimenting…without taking a minimum of risk?” (cited in Fredrickson 1991).

In contrast to the case of PEDs, however, the separation between research and use of the technology is more difficult to achieve in the case of recombinant DNA and GMOs due to the nature of the entities involved. First of all, work on recombinant DNA cannot be done without the use of microbes (most importantly bacteria). Microbes, however, are small and can be difficult to handle (accidental release being one potential issue). Also, once in a suitable environment bacteria (or other microbes such as viruses) can grow quickly and spread relatively easily. Together, these factors mean that creating and working with recombinant DNA and modified microbes potentially puts people and the environment at risk if no special measures are taken (and even if they are taken there will often be a residual element of risk that cannot be eliminated).

At the Asilomar conference researchers therefore made containment the key issue, as—in the context of recombinant DNA technology at least—it is only through containment that a safe space for further research could be created (Berg 2008; Jasanoff et al. 2015). Without this safe space it would not be possible to do further research on the risks of recombinant DNA and genetically modified microbes without violating the PP.

The Asilomar strategy therefore consists of two steps, a first step that introduces specific containment measures that aim to create a separation between research context and use context and a second step in which this safe space is used to investigate the technology or entities in question. This means that in order to be successful the Asilomar approach has to fulfil two conditions, namely (1) that the containment strategy actually works and (2) that the safe space that the containment creates allows researchers to do the right kind of experiments, i.e. experiments that can assess the potential of modified microbes/DNA to create harm.

### Did it Work?

When it comes to the question of whether the two conditions were fulfilled then the answer probably has to be a ‘yes’ in the first case and a ‘we don’t know (yet)’ in the second.

The first condition has (in principle at least) been fulfilled as containment practices are now available for experiments with modified bacteria and viruses, mostly in the form of physical containment procedures that standardise how bacteria and viruses are handled in laboratories, how contaminated glass ware and other instruments have to be treated, and so on.^19^

The second condition, however, is more difficult to assess. Clearly, there has been a lot of work done over the last few decades on the behaviour of GMOs and recombinant DNA elements [for instance the potential toxicity of genetically-modified plants for other parts of an ecosystem (Domingo and Bordonaba 2011)]. Many of these studies have shown that there are no significant negative effects of specific GMOs (relative to a particular set of measured parameters). Results like these are what led a majority of researchers to claim that there are no significant issues with the creation and the use of genetically modified microorganisms or plants (see, e.g. Berg 2008; de Lorenzo 2010; Naranjo 2014). However, critics claim that the studies performed to date have not given us conclusive answers about the safety of the technology (for instance because they have been limited in scope), meaning that even within the scientific community there still are many who are sceptical of the technology (Hilbeck et al. 2015). Importantly, some of these critics (which include practising scientists) question whether the right kind of experiments can be performed in the safe space, meaning that the debate on the second condition is still ongoing. Because of this the answer to the above question probably has to be a ‘we don’t know (yet)’.

However, in what follows this lack of consensus will be ignored as the question of interest is whether the two-step strategy, assuming it was successful, can also be applied to the CRISPR–Cas9 case.

## CRISPR–Cas9, Genome Editing and the Two-Step Asilomar Approach

As became clear in the previous section, the idea of containment is a central component of the Asilomar strategy; containment is what created the safe space that would allow researchers to perform experiments on modified DNA/organisms without risking violating the precautionary measures taken. To assess whether this strategy can be transferred to the CRISPR–Cas9 case it will therefore be central to assess (a) how containment is achieved in this case and (b) what kind of safe space it creates for experimentation.

### The Containment of Embryos: Creating a Safe Space for Experimentation

As in the Asilomar case, a key goal of the containment in the CRISPR–Cas9 case is to avoid the spreading of the DNA modifications that are introduced into the target cells. However, containment takes on a very different form here as the target of the modifications are not microbes but human cells. Importantly, the only way for a human cell to spread its modified DNA to the genome of other cells is by passing on its DNA to future generations. This can only happen if the original edit took place in germline cells *and* if these cells are then used for reproduction. Modified DNA can therefore be contained simply by prohibiting the implantation of modified embryos. This is sufficient as embryos themselves are extremely fragile and will not survive outside the laboratory environment (it is already difficult enough to get them to grow for a week or more in a petri dish). And as implantation is by definition part of clinical research, a ban on the clinical use of germline editing is enough to achieve full containment of the modifications introduced. It becomes clear, then, that the ban on clinical applications of CRISPR–Cas9 (and other genome-editing technologies) reproduces the two-step approach of the Asilomar strategy by creating a suitable containment of the modified zygotes or embryos.

The question is now whether the research space created by this containment actually allows researchers to assess the safety of the technology. To answer this question it will be crucial to go back to the beginning of this paper and look again at the uncertainties that matter in the CRISPR–Cas9 case.

### How Uncertainty Comes to Matter

When the uncertainties that surround the CRISPR–Cas9 case are compared to those of the Asilomar case some key differences in how they come to matter become apparent. As has been pointed out in “CRISPR–Cas9 and Uncertainty” section, in the discussions about CRISPR–Cas9 the precision of the modification step itself and the effect the modification has on the target organism play a central role. Interestingly, even though these uncertainties are present as well in the Asilomar case they did not shape the way researchers went about their work. The neglect of these uncertainties can be explained by the moral status ascribed to the entities that are the targets of the modifications in the Asilomar case: when experimenting with bacteria, viruses or plants the researchers did not have to worry about the lack of precision or the negative effects the modifications could have on the target organism itself because microbes and plants were not (and still are not) seen as entities with special moral status. This means that researchers could perform as many trials—and in the process waste as many organisms—as they wanted (or could afford). In the case of human embryos, however, the situation is different as the safe space for experimentation is populated by the very entity that should not be harmed.

Whether or not this is a problem of course depends on the way in which the moral status of humans is defined. If the moral status of the human being, for instance, is restricted to certain stages of its life cycle and if only stages outside of the defined range are used for experimentation then the safe space is not breached. An example of such a restriction is the well-known 14-day rule, which states that a human embryo is not an individual with its own moral status before it has reached day 14 of its development.^20^ The 14-day rule is important here as it allows researchers to do destructive/harmful research on early-stage human embryos, meaning that performing genome editing experiments on human embryos is not problematic as long as these experiments are restricted to certain developmental stages.

There is obviously an arbitrary element to the 14-day rule (why focus on the twinning event?) and over the years there were many who did not agree with it (arguing, for instance, that embryos already gain moral status before the 14-day threshold). The point here, however, is that even if the rule is accepted the problems for the Asilomar strategy would not disappear (when it is applied to the case of human germline editing). To see why that is so it is instrumental to reflect on the actual nature of the entities that are modified and how they are contained.

### The Processual Nature of Organisms

As described in “The Two-Step Asilomar Approach and the Idea of Containment” section, the containment proposed (and largely achieved) in the Asilomar case was physical containment: none of the modified organisms should be set free before the research on them showed that it is permitted to do so. In “The Containment of Embryos: Creating a Safe Space for Experimentation” section it was shown that because of the different characteristics of embryos a different containment procedure is put in place in the CRISPR–Cas9 case: what is contained are not physically bounded bodies but particular developmental stages of the organism of interest. What matters is that modified embryos are not allowed to leave a certain stage of their development, rather than a physical space. With this switch to a focus on stages the processual nature of organisms now takes centre stage, something the researchers in the Asilomar case did not have to worry about.

Dividing what is one large process into stages can of course be a useful strategy especially if the manipulation of interest is restricted to a narrow time window. This clearly is the case for the DNA modification step itself and the uncertainty about its precision. The modification is usually introduced at the zygote stage by injecting the components of the CRISPR–Cas9 system (see “The CRISPR–Cas9 system” section). The modification of genomic DNA will take place as soon as the Cas9 protein is produced and a functional complex with the guide RNA is formed. If an error occurs (i.e. an off-target site is modified) it will happen at this stage. To do further research to overcome the uncertainty of this modification step it is therefore sufficient to work with zygotes and early-stage embryos—other stages of development simply don’t matter as the uncertainty is limited to an event that happens directly after the injection step. In this case, then, the ban (in combination with the 14-day rule) indeed creates a safe space for researchers to investigate and further optimise the modification step of genome editing.

The same, however, is not the case for the second uncertainty discussed in “CRISPR–Cas9 and Uncertainty” section, i.e. the worry about the effects the modification could have on the organism as a whole. Clearly, in this case the processual nature of organisms means that in order to get the full picture of the potential harm of a genetic modification for its carrier scientists would have to follow the human embryo all the way through its development to adulthood. The uncertainty in question does not have to do with the particulars of the CRISPR–Cas9 system but with the effects the newly introduced modifications have on the organism. These effects are not only systemic but can also affect any stage of the life cycle of the organism. This means that in order to overcome the uncertainty about these effects researchers would have to test all stages of the ongoing life cycle, which ultimately means that modified embryos would have to be implanted and allowed to fully develop into adult human beings. Such a course of action clearly violates the containment that forms the essence of the two-step strategy.

### Model Organisms and New Rules to the Rescue?

A likely objection here is that the problem could be solved by working with model organisms instead of human embryos. This is after all a strategy that Baltimore and colleagues also suggest in their discussion of how scientists should proceed when studying the potential dangers of the CRISPR–Cas9 system (Baltimore et al. 2015).

But how realistic is such a replacement of the human subject with model organisms when assessing the second uncertainty? It is interesting to note here that in the case of GMO safety assessments (for instance in the analysis of GM plant toxicity) the ultimate test is always the field test: without tests that use actual modified plants in their fully grown and functional states the risk assessment is simply not complete.^21^ The same applies to the approval procedure for novel medical treatments where clinical trials on human subjects are required.

Given this focus on testing the actual modified and fully-grown organism in the case of GM plants and medical treatments it is not clear why in the case of germline editing it should suddenly be sufficient to analyse the effect of a modification on, for instance, mice but not humans. Model organisms might of course be used (as they are) to get an initial idea of what effects a particular modification could have on the development and/or functioning of the organism, but the final test will always be to assess the effects on the actual organism of interest.^22^

Another solution would be to change the 14-day rule and to allow researchers to experiment on embryos in later stages of development.^23^ This would mean that the effects of newly introduced genomic modifications could be assessed for later stages of development (which could be a significant benefit as key structures of the organism are only formed after day 14).^24^

But whilst an extension of the 14-day rule would certainly allow for a somewhat more comprehensive assessment of the risks a particular genomic modification poses, it would represent a limited risk analysis. The point here is that the organism is an ongoing process and development is not something that is restricted to the first two or four weeks after fertilization. There are many junctures at almost any stage of a human life cycle that matter for the proper functioning of the human body and at which the modified DNA might unfold its effects. This means that many of the potential dangers (or benefits) of a particular modification could not be assessed unless the embryo is implanted and allowed to develop into an adult being.

The point here is not to say that doing more research on (early-stage) human embryos and/or model organisms won’t help in learning more about the entities and processes involved. The point is rather to highlight that doing the research that is needed to assess the full range of uncertainties that drove the calls for precautionary measures will mean that the PP has to be violated in one way or the other. This is important as the soothing promises of the Asilomar ban—“There is a safe and responsible way of doing more research on new molecular tools”—falls apart in the CRISPR–Cas9 case. The research that needs to be done to address the uncertainties cannot be done in a safe space, as the two conditions (creating a contained space and doing research to assess the dangers of the technology) clash with each other. The release of the modified human embryos will have to be part and parcel of the safety assessment, because the whole life cycle of the organism will have to be assessed to get an insight into the potential dangers (and benefits) each modification carries for the organism as a whole. What this means is that the science involved in developing the new molecular tool is inherently risky (something that is not surprising given that science is always moving at the frontier of what is known and what can be done). This, however, also means that the ‘prudent way forward’ that researchers have proposed is less safe than it might seem at first. And with this it loses its main appeal as a trust-building exercise.

## Conclusion

The goal of this paper has been to understand whether the self-imposed ban on human germline editing and the use of CRISPR–Cas9 can deliver on its promises. The question of whether this ban can work is crucial as it is used as a tool to gain or maintain the trust of the broader public (including policy-makers) in the scientists’ ability to self-regulate.

To analyse this ban it was important to first understand what this ‘prudent way forward’ actually consists of. The analysis of the precedent on which the current ban is modelled—the well-known Asilomar ban on recombinant DNA technology—has shown that it is a particular two-step approach that researchers use as the centrepiece of their strategy. This two-step strategy is composed of a containment step that should (1) create a defined space in which then (2) safe research on the risks of the method can be performed.

However, the analysis has shown that this two-step approach cannot be fully applied to the CRISPR–Cas9 case. Even though it allows the creation of a safe space for experimentation on one of the uncertainties that surround the system (i.e. the precision of the modification step), it fails when it comes to the second form of uncertainty—the lack of knowledge about the systemic effects a particular DNA modification might have. The reason for this is that the original Asilomar strategy relies on a containment step that cannot handle something that is both a process and has a moral status (a dual condition that was not fulfilled in the Asilomar case).^25^ The failure means that the safe or ‘prudent’ way forward is not as safe as it seems at first. This also affects its power as a trust-building measure.

A general insight that can be derived from this case is that it is crucial to take into account the ontology of the entities/processes of interest when discussing specific policy measures regarding novel biological tools or technologies; decisions on how to address specific risks associated with the use of new tools/technologies cannot be taken without also taking ontological issues into account. The ban on clinical applications of CRISPR–Cas9 serves as a powerful example of this: the proposed strategy (two-step containment) seems like a good idea as long as only a superficial comparison between it and its precedent is made. Upon closer inspection, however, cracks start to appear as the processual nature of organisms—which did not matter in the Asilomar case—now suddenly becomes a stumbling block for the proposed strategy. How the nature of organisms (or of other biological entities such as macromolecules) is conceptualised is important because it shapes the path that is taken regarding regulation and safety measures. And in cases like the ban on genome editing it matters whether or not researchers and policy-makers are on the right path, as the path and its potential for success is the central element that should help to preserve (or gain) the public’s trust in science.

## Footnotes

1 These are just two relatively prominent examples of research on human embryos involving the CRISPR-Cas9 system. Given the speed at which genome editing projects emerge and develop (targeting a range of organisms, from bacteria to mice, zebrafish and plants) it is not possible to offer a comprehensive and up-to-date review of the field in the context of this paper.

2 See for instance (Zimmerman 1991) and (Lappé 1991) or also (Frankel and Chapman 2000) for some examples. For a history of gene therapy see (Wirth et al. 2013).

3 Palindromic repeats are DNA sequences that read the same from both directions.

4 ‘Cas9′ is the name of a specific nuclease found in particular bacteria. Each bacterial strain contains its own set of Cas proteins and CRISPR sites to form its own CRISPR system.

5 Note that the system can not only provide protection against bacteriophages but also other mobile DNA elements, such as plasmids.

6 Note that injection of RNA is not the only method of getting the CRISPR-Cas9 components into a target cell.

7 For a more detailed history of the discovery and an overview of the biological function of the CRISPR system see (Pennisi 2013; Doudna and Charpentier 2014; Morange 2015).

8 Note that this brings some terminological issues with it. As the editing of genomic DNA does not necessarily mean that foreign DNA sequences are introduced (as researcher could now simply change the sequence of an existing gene to an allelic form found in other organisms of the same species) it is not clear whether edited organisms should be classified the same as organisms that contain recombinant DNA (i.e. what is traditionally called a ‘genetically-modified organism’ or ‘GMO’) (Ainsworth 2015). This obviously has important consequences for how edited organisms should be regulated. For the purposes of this paper, however, GMOs and edited organisms will be treated as the same, as the uncertainties that are the key focus here affect both cases.

9 Note that the focus here is only on uncertainties as (narrowly) defined by scientists (see “Introduction” section).

10 Note that there is no general rule regarding the percentage of sequence identity that is required for functional complexes to be formed.

11 See for instance the results of (Liang et al. 2015) for some important caveats on the alleged precision of the system. See also the discussion of off-target effects in (Ledford 2015).

12 See: http://www8.nationalacademies.org/onpinews/newsitem.aspx?RecordID=12032015a for the summary statement of the organizing committee (many of whom were also co-authors of one of the earlier calls for a ban (Baltimore et al. 2015)).

13 This again illustrates how the two issues of a ban on germline editing and a ban on CRISPR-Cas9 are intermixed in these discussions.

14 Or as Lanphier and colleagues put it: “In our view, genome editing in human embryos using current technologies could have unpredictable effects on future generations. This makes it dangerous and ethically unacceptable. […] At this early stage, scientists should agree not to modify the DNA of human reproductive cells” (Lanphier et al. 2015, p. 410). See also (Harris 2015; Sugarman 2015) or the 2015 Hinxton Group statement (http://www.hinxtongroup.org/Hinxton2015_Statement.pdf) for similar positions.

15 See for instance the so-called ‘Wingspread Statement on the Precautionary Principle (www.who.int/ifcs/documents/forums/forum5/wingspread.doc): “When an activity raises threats of harm to human health or the environment, precautionary measures should be taken even if some cause and effect relationships are not fully established scientifically.”

16
http://www.easa.europa.eu/newsroom-and-events/press-releases/easa-allows-electronic-devices-remain-and-connected-throughout.

17 See, for instance, http://www.boeing.com/commercial/aeromagazine/aero_10/interfere_textonly.html.

18 Note that scientists were not worried about the negative effects recombinant DNA might have on the modified organisms themselves. This is in stark contrast to the situation in human genome editing (“CRISPR-Cas9, Genome Editing and the Two-Step Asilomar Approach” section).

19 Many of these measures were already known and in use in the 1970 s and formed part of the recommendations made at the Asilomar conference (Berg et al. 1975).

20 The reason for this particular cut-off is that an embryo could still be split into two viable entities (or be fused with another embryo) until around day 14, meaning it is, in principle at least, not one specific individual yet. The 14-day rule was first proposed in 1979 in the US and then applied in 1984 in the Warnock report (http://www.hfea.gov.uk/2068.html).

21 The European guidelines for the environmental risk assessment of GM plants prescribe a hierarchy of experiments that need to performed, starting with laboratory trials but ultimately ending in field trials (Hilbeck et al. 2011).

22 There is a considerable literature in philosophy of science on the use of model organisms in the sciences, see, for instance (Burian 1993; Bolker 1995; Ankeny 2001; Schaffner 2001; Piotrowska 2013).

23 Note that such an extension of the 14-day rule has recently been proposed, partly in response to new technological advances that make the in vitro cultivation of human embryos possible past the current threshold of about nine days (see (Hyun et al. 2016)).

24 One example is gastrulation, i.e. the formation of the three main tissue layers of the organism, an event that usually starts after day 14 of normal development.

25 There are of course further reasons why the Asilomar strategy might fail, for instance because of the way in which the scientific environment has changed since the days when the Asilomar ban was put in place. Today research is a much more global affair with even more (and more diverse) players involved than was already the case in the 1970 s. These quantitative differences might well make a qualitative difference in the way we have to approach the regulation of the (molecular) life sciences. I thank E. Wyler for pointing out this aspect of the issue. See also (Nature Editorial 2015) for a similar view.
